# Implications of helplessness in depression: diagnosing mild cognitive impairment and analyzing its effects on cognitive decline in older adults

**DOI:** 10.3389/fnagi.2024.1378676

**Published:** 2024-05-27

**Authors:** Boung Chul Lee, Young Min Choe, Guk-Hee Suh, Musung Keum, Shin Gyeom Kim, Hyun Soo Kim, Jaeuk Hwang, Dahyun Yi, Jee Wook Kim

**Affiliations:** ^1^Department of Neuropsychiatry, Hallym University Hangang Sacred Heart Hospital, Seoul, Republic of Korea; ^2^Department of Psychiatry, Hallym University College of Medicine, Chuncheon, Republic of Korea; ^3^Department of Neuropsychiatry, Hallym University Dongtan Sacred Heart Hospital, Hwaseong, Republic of Korea; ^4^Department of Neuropsychiatry, Soonchunhyang University Bucheon Hospital, Bucheon, Republic of Korea; ^5^Department of Laboratory Medicine, Hallym University Dongtan Sacred Heart Hospital, Hwaseong, Republic of Korea; ^6^Department of Psychiatry, Soonchunhyang University Hospital Seoul, Seoul, Republic of Korea; ^7^Institute of Human Behavioral Medicine, Medical Research Center Seoul National University, Seoul, Republic of Korea

**Keywords:** helplessness, depression, mild cognitive impairment, Alzheimer’s disease, cognitive decline

## Abstract

**Background:**

This study focuses on how elements of depression correlate with mild cognitive impairment (MCI) in older adults and the diagnostic efficacy of combining these components with the Mini-Mental State Examination (MMSE). The study also investigated the connection between individual depression components and overall cognitive function, as measured by the total score (TS) of the consortium to establish a registry for Alzheimer’s disease (AD) assessment battery.

**Methods:**

The study included 196 nondemented adults aged 65 to 90 years at a university hospital and community. Comprehensive clinical assessments including the 30-item Geriatric Depression Scale (GDS) to measure components of depressive symptoms, TS, and blood nutritional biomarkers.

**Results:**

Our stepwise logistic regression analysis highlighted the ‘helplessness item’ (odds ratio = 4.531, 95% CI = 2.218 to 9.258, *p* < 0.001) as a significant predictor for MCI diagnosis. Further, models incorporating ‘helplessness item + MMSE’ demonstrated markedly enhanced accuracy in diagnosing MCI, surpassing the performance of the MMSE used independently. Notably, the group characterized by helplessness showed a significant reduction in TS (B = −5.300, SE = 1.899, β = −0.162, *p* = 0.006), with this trend being particularly pronounced in individuals exhibiting lower levels of physical activity. Interestingly, this correlation did not manifest in participants with higher physical activity levels.

**Conclusion:**

Our findings suggest that helplessness is highly effective in diagnosing MCI and is linked to a decrease in cognitive function. Therefore, when addressing MCI and AD-related cognitive decline, clinicians should consider helplessness.

## Introduction

Depressive symptoms are commonly linked to Alzheimer’s disease (AD) and related cognitive decline in older adults (Jorm, 2000; Milwain and Nagy, 2005; Barnes et al., 2006; Byers and Yaffe, 2011; Bennett and Thomas, 2014; Bellou et al., 2017; Harerimana et al., 2022). Prospective epidemiological studies have demonstrated that depressive symptoms are associated with an increased risk of AD across all age groups (Jorm, 2000; Barnes et al., 2006; Byers and Yaffe, 2011; Bennett and Thomas, 2014; Bellou et al., 2017). A molecular study has uncovered a shared genetic basis between depressive symptoms and AD, providing genetic evidence supporting the causal role of depression in AD (Harerimana et al., 2022). Furthermore, a post-mortem study indicated that depressive symptoms heighten the probability of cognitive impairment in older adults with subclinical AD pathology (Milwain and Nagy, 2005). It is noteworthy that the majority of studies investigating the relationship between depression and AD used the cumulative sum of overall symptoms of depression or the diagnosis of depressive disorder, not individual depressive components, to represent depression (Jorm, 2000; Milwain and Nagy, 2005; Barnes et al., 2006; Byers and Yaffe, 2011; Bennett and Thomas, 2014; Bellou et al., 2017; Harerimana et al., 2022).

To understand the exact nature of the association of depression with AD and related cognitive decline, it is essential to investigate whether the individual’s depressive components, as well as the overall symptoms of depression, influence the association. Nevertheless, there is scant evidence indicating links between individual components of depression, including emotional or subjective cognitive disturbances, and AD and related cognitive decline.

Overall, it is crucial to investigate the diagnostic accuracy of depression components in detecting prodromal AD, namely mild cognitive impairment (MCI), to accurately understand the impact of depression on AD and cognitive decline in older adults. MCI serves as transitional phase between normal aging and dementia, with estimated prevalence in the general older population ranging from 5.0 to 36.7% (Sachdev et al., 2015). Notably, 11 to 33% of individuals with MCI progress to dementia within 2 years (Luis et al., 2003; Bruscoli and Lovestone, 2004). Consequently, MCI is thus recognized as significant risk factor for dementia, particularly AD (Luis et al., 2003; Bruscoli and Lovestone, 2004).

Hence, our study aimed to explore two main areas: First, we examined how individual elements of depression relate to the diagnosis of MCI in older adults. Secondly, we compared the accuracy of diagnosing MCI when combining these depression components with the Mini-Mental State Examination (MMSE) against using the MMSE alone. In addition, we investigated the connection between individual depression components and overall cognitive function, as measured by the total score (TS) of the consortium to establish a registry for AD (CERAD) assessment battery.

## Materials and methods

### Participants

This study is a component of the General Lifestyle and AD (GLAD) study, an ongoing prospective cohort study launched in 2020. By January 2022, the study had recruited 225 adults for eligibility assessment. Out of these, 196 non-demented individuals aged between 65 and 90 years were enrolled in the initial GLAD cohort. The exclusion of 29 participants was based on the following criteria: 7 were disqualified due to either existing comorbid conditions—medical, psychiatric, or neurological—that could influence mental functioning (5 individuals), severe communication or behavioral issues complicating clinical assessments (1 individual), or concurrent participation in another clinical trial involving an investigational product (1 individual). Additionally, 2 participants were excluded for not fitting any diagnostic categories, and 20 were removed from the study due to either withdrawal of consent (17 individuals) or loss of contact (3 individuals). The composition of the baseline GLAD cohort included 113 cognitively normal (CN) adults and 83 individuals diagnosed with mild cognitive impairment (MCI). Eligible participants were identified through the dementia screening program conducted at the memory clinic of Hallym University Dongtan Sacred Heart Hospital in Hwaseong, South Korea. Moreover, community volunteers were enlisted through recommendations from existing participants, family members, friends, or acquaintances. The CN individuals had Clinical Dementia Rating (CDR) (Morris, 1993) score of 0 and no diagnosis of MCI or dementia. The MCI individuals had CDR of 0.5 and met the inclusion criteria outline in the core clinical criteria for diagnosis of MCI, as per the recommendations of the NIA-AA guidelines (Albert et al., 2011). Regarding objective memory impairment, the age-, education-, and sex-adjusted z-score was < −1.0 for at least one of four episodic memory tests included in the Korean version of the CERAD-K neuropsychological battery: word list memory (WLM), word list recall (WLR), word list recognition (WLRc), and constructional recall (CR) tests (Morris et al., 1989; Lee et al., 2002, 2004). The exclusion criteria included the existence of a major psychiatric disorder, a notable neurological or medical condition, or any coexisting health issue capable of influencing mental function. Additionally, factors such as illiteracy, visual or hearing impairments, severe communication or behavioral issues that could hinder clinical examinations, and the utilization of an investigational drug were considered grounds for exclusion.

### Clinical assessments

Each participant underwent standardized clinical assessments by trained psychiatrists following the clinical assessment of the GLAD study protocol. This protocol included the Korean version of the CERAD (CERAD-K) (Morris et al., 1989; Lee et al., 2002). Additionally, trained neuropsychologists administered the GLAD neuropsychological assessment protocol, which incorporated the CERAD-K neuropsychological battery (Lee et al., 2004) to all participants. To evaluate the relationship between individual items of depression and overall cognitive function, we utilized the total score (TS) of the CERAD (Seo et al., 2010). This score, indicative of overall cognitive ability, is derived by totaling the scores from seven tests within the CERAD neuropsychological battery, including verbal fluency, the Boston Naming Test, WLM, constructional praxis, WLR, WLRc, and CR tests. In comparing the diagnostic accuracy of individual depression items for MCI, we utilized the MMSE (Nakata et al., 2009), one of the most widely used scales in clinical settings, either alone or in combination with individual depression items. The evaluation of vascular risk factors (VRF), such as hypertension, diabetes mellitus, dyslipidemia, coronary heart disease, transient ischemic attack, and stroke, involved systematic interviews of participants and their family members, with data collection performed by trained researchers. The vascular risk score (VRS) was determined by calculating the percentage of present VRF (DeCarli et al., 2004). To assess the severity and components of depressive symptoms, the 30-item Geriatric Depression Scale (GDS) was employed, with detail available at https://web.stanford.edu/~yesavage/GDS.english.long.html (Yesavage et al., 1982; Kim et al., 2008). The body mass index (BMI) was computed by dividing the body weight in kilograms by the square of the height in meters, following the guidelines of the World Health Organization (WHO) available at https://www.euro.who.int/en/health-topics/disease-prevention/nutrition/a-healthy-lifestyle/body-mass-index-bmi. Physical activity was measured using the Korean-version of the Physical Activity Scale for the Elderly (PASE) (Washburn et al., 1993; Choe et al., 2010). The PASE score, indicating the level of physical activity, was calculated by summing weighted scores from leisure, household, and occupational activity subscales. Participants’ activity levels were scored and divided into low and high categories, with higher scores indicating greater activity. The study divided participants into three income-based groups: those earning less than the minimum cost of living (MCL), those earning between the MCL and twice the MCL, and those earning at least twice the MCL, as defined by the Ministry of Health and Welfare, Republic of Korea, in November 2012.^1^ The MCL for a single-person household was set at 572,168 Korean Won per month (approximately US$ 507.9), with an additional 286,840 Korean Won (about US$ 254.6) per month added for each extra member in the household. Lifetime alcohol intake status (never/former/drinker) and smoking status (never/ex-smoker/smoker) were assessed through interviews conducted by trained researchers and a review of medical records. To ensure the accuracy of information, reliable informants were interviewed.

### Measuring blood biomarkers

Following an overnight fast, blood samples were collected via venipuncture in the morning (08,00–09:00). Albumin, glucose, high-density lipoprotein (HDL)-cholesterol, and low-density lipoprotein (LDL)-cholesterol were determined using a COBAS c702 analyzer and dedicated reagents (Roche Diagnostics, Mannheim, Germany). Apolipoprotein E (apoE) was genotyped using a Seeplex ApoE ACE genotyping kit (Seegene, Seoul, Korea). The positivity for the apoE ε4 allele (APOE4) was defined as the presence of at least one ε4 allele.

### Statistical analyses

Between-group comparisons for continuous data, encompassing demographic and clinical information, were conducted using two-tailed *t*-tests. Categorical data were analyzed through the chi-square test. Logistic regression analysis was performed to identify specific items within the 30-item GDS that could predict MCI. Stepwise logistic regression analyses were executed to pinpoint the GDS items minimizing the probability of misclassification between subjects with and without MCI, utilizing the likelihood ratio statistic for item entry and removal at *p* < 0.05 and *p* < 0.10. To assess the diagnostic accuracy of components of depression along with MMSE, multiple logistic regression analyses were performed with GDS-items and MMSE as the independent variables and MCI status as the dependent variable. Given potential confounders such as VRS, BMI, PASE, alcohol intake, smoking, and blood nutritional markers, including albumin, glucose, HDL- and LDL-cholesterol, all participants were systematically evaluated. Two models were tested, adjusting for covariates in a stepwise fashion. The first model included age, sex, education, APOE4, and VRS as covariates. The second model encompassed these covariates plus BMI, PASE, alcohol intake, smoking, albumin, glucose, and HDL- and LDL-cholesterol. We utilized the differences in −2 log likelihood (−2LL) for the statistical comparison of the predictive ability among various models with different numbers of independent variables (Lee et al., 2006). The −2LL is a measure derived from logistic regression analysis and is directly correlated with the contribution of variables to the distinction between groups; a smaller −2LL value suggests a superior predictive ability of the model. The probability distribution of −2LL difference between a simple (model 1) and a more complex model (model 2) can be approximated by a chi-square distribution with the difference between degrees of freedom ( Δ *df* = *df*2 − *df*1), where *df*1 and *df*2 represent the degrees of freedom of models 1 and 2, respectively. Consequently, the difference in −2LL enables a direct comparison of prediction models with different complexities (Hosmer, 1989). As part of sensitivity analyses, the same analyses were conducted for subjects exhibiting no moderate to extreme severe depressive symptoms (GDS score ≤ 23), employing a cutoff score derived from prior research (Yi, 2016). This approach was adopted as depression might exhibit greater discriminatory effects in individuals with MCI. To examine the relationship between the individual depression items, which were deemed significant in the aforementioned analyses, and overall cognitive function, we conducted regression analysis. In this analysis, each individual depression item was used as an independent variable and overall cognitive function as the dependent variable. Covariates were controlled using the two-step model approach as described above. Moreover, we explored the moderation effects of age, sex, APOE4, education, GDS, VRS, BMI, and PASE status on the relationships between the individual depression items and the overall cognitive function. We included two-way interaction term between the individual depression item and any one of the factors, as well as the individual depression item itself, as an independent variable in the regression model. For significant interactions, subsequent subgroup analyses were performed using an additional regression model for each subgroup divided by the moderation variable. All statistical analyses were carried out using SPSS Statistics software ver. 28 (IBM, Armonk, NY, United States).

### Ethics statement

The current study protocol underwent review and approval by the institutional review board of Hallym University Dongtan Sacred Heart Hospital and was conducted in accordance with the principles outlined in the current version of the Declaration of Helsinki. Participants provided informed consent to participate in the study.

## Results

### Participants

The demographic and clinical characteristics of the study population are presented in Tables 1, 2. Among the 196 nondemented adult participants, 113 were classified as CN, and 83 were diagnosed with MCI.

**Table 1:** Participant characteristics according to clinical diagnosis status.

Characteristic	Overall	CN	MCI	*p*
*n*	196	113	83	
Age, y	72.65 (5.95)	71.88 (5.46)	73.70 (6.45)	0.035^a^
Female, *n* (%)	113 (57.65)	83 (73.45)	30 (36.14)	0.276^b^
Education, y	9.62 (4.51)	9.82 (4.53)	9.35 (4.49)	0.469^a^
MMSE score	25.58 (3.45)	26.63 (2.54)	24.16 (3.99)	<0.001^a^
APOE4-positivity, *n* (%)	39 (19.90)	15 (13.27)	24 (28.92)	0.007 ^b^
VRS, %	23.98 (18.58)	24.34 (18.23)	23.49 (19.13)	0.755^a^
BMI	24.83 (3.41)	24.72 (2.95)	24.98 (3.96)	0.591^a^
Annual income, *n* (%)				0.012^b^
<MCL	25 (0.13)	12 (10.62)	13 (15.66)	
≥ MCL, <2 × MCL	62 (31.63)	28 (24.78)	34 (40.96)	
≥ 2 × MCL	109 (55.61)	73 (64.60)	36 (43.37)	
UPDRS, gait disturbance requiring assistance	0 (0.00)	0 (0.00)	0 (0.00)	
Physical activity				
Total score of PASE	64.77 (46.21)	68.93 (47.70)	59.11 (43.74)	0.142^a^
Alcohol drink status, *n* (%)				0.092^b^
Never	107 (54.59)	69 (61.06)	38 (45.78)	
Former	34 (17.35)	18 (15.93)	16 (19.28)	
Drinker	55 (28.06)	26 (23.01)	29 (34.94)	
Smoking status, *n* (%)				0.101^c^
Never	149 (76.02)	92 (81.42)	57 (68.67)	
Former	39 (19.90)	17 (15.04)	22 (26.51)	
Smoker	8 (4.08)	4 (3.54)	4 (4.82)	
Blood nutritional markers				
Albumin, g/dL	4.57 (0.26)	4.60 (0.26)	4.53 (0.26)	0.057^a^
Glucose, fasting, mg/dL	108.15 (19.94)	107.77 (20.13)	108.67 (19.80)	0.756^a^
HDL-cholesterol, mg/dL	54.64 (12.96)	55.04 (12.10)	54.11 (14.10)	0.624^a^
LDL-cholesterol, mg/dL	96.41 (33.82)	98.53 (32.98)	93.51 (34.94)	0.309^a^
GDS				
Total score	10.92 (7.24)	9.81 (6.85)	12.42 (7.53)	0.012^a^
Individual item, n (%)				
item #10 (helpless)	69 (35.20)	28 (24.78)	41 (49.40)	<0.001^b^
item #14 (subjective memory problems)	70 (35.71)	33 (29.20)	37 (44.58)	0.026^b^
CERAD cognition				
Total score	69.98 (15.61)	77.14 (11.93)	60.23 (14.77)	<0.001

MMSE, mini-mental state examination; APOE4, apolipoprotein E ε4 allele; VRS, vascular risk score; BMI, body mass index; MCI, mild cognitive impairment; MCL, minimum cost of living; UPDRS, Unified Parkinson’s Disease Rating Scale; PASE, physical activity scale for the elderly; GDS, geriatric depression scale; CERAD, Consortium to Establish a Registry for Alzheimer’s Disease.

Data are expressed as mean (standard deviation), unless otherwise indicated.

a by one-way analysis of variance.

b by chi-square test.

c by fisher exact test.

**Table 2:** Participant characteristics according to helplessness status.

Characteristic	Overall	Non-helplessness	Helplessness	*p*
*n*	196	127	69	
Age, y	72.65 (5.95)	72.54 (5.79)	72.86 (6.29)	0.727^a^
Female, *n* (%)	113 (57.65)	90 (70.87)	48 (69.57)	0.849^b^
Education, y	9.62 (4.51)	9.80 (4.56)	9.30 (4.42)	0.468^a^
MMSE score	25.58 (3.45)	25.77 (3.43)	25.23 (3.48)	0.297^a^
APOE4-positivity, *n* (%)	39 (19.90)	29 (22.83)	10 (14.49)	0.162^b^
VRS, %	23.98 (18.58)	24.02 (18.38)	23.91 (19.06)	0.971^a^
BMI	24.83 (3.41)	24.94 (3.35)	24.63 (3.52)	0.545^a^
MCI, *n* (%)	83 (42.35)	42 (33.07)	41 (59.42)	<0.001
Annual income, *n* (%)				0.014^b^
<MCL	25 (0.13)	12 (0.08)	13 (18.84)	
≥ MCL, <2 × MCL	62 (31.63)	35 (27.56)	27 (39.13)	
≥ 2 × MCL	109 (55.61)	80 (62.99)	29 (42.02)	
UPDRS, gait disturbance requiring assistance	0 (0.00)	0 (0.00)	0 (0.00)	
Physical activity				
Total score of PASE	64.77 (46.21)	66.27 (47.64)	62.03 (43.65)	0.541^a^
Alcohol drink status, *n* (%)				0.705^b^
Never	107 (54.59)	70 (55.12)	37 (53.62)	
Former	34 (17.35)	20 (15.75)	14 (20.29)	
Drinker	55 (28.06)	37 (29.13)	18 (26.09)	
Smoking status, *n* (%)				0.266^c^
Never	149 (76.02)	101 (79.53)	48 (69.57)	
Former	39 (19.90)	21 (16.54)	18 (26.09)	
Smoker	8 (4.08)	5 (3.94)	3 (4.35)	
Blood nutritional markers				
Albumin, g/dL	4.57 (0.26)	4.58 (0.26)	4.56 (0.27)	0.740^a^
Glucose, fasting, mg/dL	108.15 (19.94)	109.51 (21.55)	105.68 (16.50)	0.201^a^
HDL-cholesterol, mg/dL	54.64 (12.96)	55.20 (12.74)	53.64 (13.38)	0.423^a^
LDL-cholesterol, mg/dL	96.41 (33.82)	94.46 (47.64)	99.94 (36.16)	0.281^a^
GDS total score	10.92 (7.24)	7.10 (4.36)	17.94 (6.17)	<0.001^a^
CERAD cognition				
Total score	69.98 (15.61)	72.24 (15.51)	65.83 (15.05)	0.006^a^

MMSE, mini-mental state examination; APOE4, apolipoprotein E ε4 allele; VRS, vascular risk score; BMI, body mass index; MCI, mild cognitive impairment; MCL, minimum cost of living; UPDRS, Unified Parkinson’s Disease Rating Scale; PASE, physical activity scale for the elderly; GDS, geriatric depression scale; CERAD, Consortium to Establish a Registry for Alzheimer’s Disease.

Data are expressed as mean (standard deviation), unless otherwise indicated.

a by one-way analysis of variance.

b by chi-square test.

c by fisher exact test.

### Selection of predictive GDS items for MCI diagnosis

Following the models obtained through stepwise logistic regression analyses, GDS item #10 (helplessness: “Do you often feel helpless?”; odds ratio = 4.531, 95% CI = 2.218 to 9.258, *p* < 0.001) and GDS item #14 (subjective memory problems: “Do you feel you have more problems with memory than most?”; odds ratio = 1.999, 95% CI = 1.040 to 3.840, *p* = 0.038) were extracted from the 30-item GDS as having significant contributions to the diagnostic accuracy for MCI (Table 3).

**Table 3:** Multiple logistic regression analyses of helplessness and subjective memory problems for MCI diagnosis.

	OR	95% CI	*p*	R^2^
GDS item #10 (helplessness)				
Model 1^a^	3.692	1.911 to 7.133	<0.001	0.198
Model 2^b^	4.531	2.218 to 9.258	<0.001	0.269
GDS item #14 (subjective memory problems)				
Model 1^a^	1.993	1.057 to 3.756	0.033	0.128
Model 2^b^	1.999	1.040 to 3.840	0.038	0.166

GDS, geriatric depression scale; MCI, mild cognitive impairment; APOE4, apolipoprotein E ε4 allele; VRS, vascular risk score; BMI, body mass index.

a Adjusted for age, sex, education, APOE4, and VRS.

b Adjusted for age, sex, education, APOE4, VRS, BMI, annual income, physical activity, alcohol intake, smoking, albumin, fasting glucose, and HDL-/LDL-cholesterol.

### Impact of GDS items alone and in combination with MMSE on MCI diagnosis accuracy

Initially, helpless item (GDS item #10) and subjective cognitive problem item (GDS item #14) (referencing the results from the aforementioned stepwise logistic regression analyses), along with MMSE (recognized as one of the best objective cognitive tools for predicting MCI diagnosis), were selected as candidate independent variables for logistic regression analyses to compare models. The analyses proceeded in three steps (Table 4). In the first step, three one-candidate models were tested: Model H (including helpless item); Model S (including subjective memory problems item); and Model M (including MMSE). Two one-candidate models, specifically Model H and Model M, demonstrated statistical significance, with Model M exhibiting the highest classification accuracy and the smallest −2LL among the three models. Additionally, Model H displayed the highest classification accuracy and the smallest −2LL among the two one-candidate models with a depression component. In the second step, the −2LL was compared between each one-candidate model and the corresponding two-candidate models, which included MMSE and either helpless item or subjective memory problems item. The MCI diagnostic accuracy of both Model HM (helplessness + MMSE) including helpless item and MMSE, and Model SM (subjective memory problems + MMSE) including subjective memory problems item and MMSE, was significantly superior to that of Model M (MMSE), which had the highest classification accuracy and smallest −2LL among the three one-candidate models. In the third step, the three-candidate model, Model HSM (helplessness + subjective memory problems + MMSE), including all three variables, was compared with Model HM or Model SM. Model HSM was significantly superior to Model SM, but not to Model HM. The ultimately selected logistic regression model for MCI diagnosis was Model HM (helplessness + MMSE) (Table 5). These findings were corroborated by a sensitivity analysis performed after excluding participants with no moderate to extremely severe depressive symptoms (Table 6).

**Table 4:** Logistic regression analyses^a^ to select appropriate models for MCI diagnosis.

	Classification accuracy (%)	−2LL	*χ*^2^	*p*	∆ *df*	Significance test for −LL difference
*One candidate model*						
Model H (helplessness)						
GDS item #10	70.1	220.967	43.316	<0.001	1	
Model S (subjective memory problems)						
GDS item #14	68.0	237.085	27.085	0.008	1	
Model M						
MMSE	71.1	213.314	50.970	<0.001	1	
*Two candidate model*						
Model HM (helplessness + MMSE)						
GDS item #10 + MMSE	78.4	192.565	71.719	<0.001	1	*Model HM vs. M: p* < 0.001 △ accuracy (%) = 7.3
Model SM (subjective memory problems + MMSE)						
GDS item #14 + MMSE	74.7	209.336	54.948	<0.001	1	*Model SM vs. M:* *p* = 0.0461 △ accuracy (%) = 3.6
*Three candidate model*						
Model HSM (helplessness + subjective memory problems + MMSE)						
GDS item #10 + GDS item #14 + MMSE	78.9	192.342	71.941	<0.001	1	*Model HSM vs. HM:* *p* = 0.6368 △ accuracy (%) = 0.5
					1	*Model HSM vs. SM:* *p* < 0.001 △ accuracy (%) = 4.2
					2	*Model HSM vs. M:* *p* < 0.001 △ accuracy (%) = 7.8

MCI, mild cognitive impairment; GDS, geriatric depression scale; MMSE, mini-mental state examination.

−2LL indicates −2 log likelihood.

a Adjusted for age, sex, education, APOE4, VRS, BMI, annual income, physical activity, alcohol intake, smoking, albumin, fasting glucose, and HDL-/LDL-cholesterol.

**Table 5:** The ultimately selected regression analyses of combined helpless item and MMSE for MCI diagnosis.

	OR	95% CI	*p*	R^2^
*Model HM (helplessness + MMSE)*				
Model 1^a^				0.349
GDS item #10	4.078	1.986 to 8.373	<0.001	
MMSE	0.725	0.632 to 0.832	<0.001	
Model 2^b^				0.415
GDS item #10	5.392	2.431 to 11.959	<0.001	
MMSE	0.711	0.616 to 0.820	<0.001	

GDS, geriatric depression scale; MCI, mild cognitive impairment; APOE4, apolipoprotein E ε4 allele; VRS, vascular risk score; MMSE, mini-mental state examination.

a Adjusted for age, sex, education, APOE4, and VRS.

b Adjusted for age, sex, education, APOE4, VRS, BMI, annual income, physical activity, alcohol intake, smoking, albumin, fasting glucose, and HDL-/LDL-cholesterol.

**Table 6:** The ultimately selected regression analyses of combined helpless item and MMSE for MCI diagnosis with no moderate to extreme severe depressive symptoms (*n* = 181).

	OR	95% CI	*p*	R^2^
*Model HM (helplessness + MMSE)*				
Model 1^a^				0.366
Helplessness	4.499	2.038 to 9.932	<0.001	
MMSE	0.728	0.631 to 0.840	<0.001	
Model 2^b^				0.425
Helplessness	6.132	2.543 to 14.788	<0.001	
MMSE	0.707	0.608 to 0.822	<0.001	

GDS, geriatric depression scale; MCI, mild cognitive impairment; APOE4, apolipoprotein E ε4 allele; VRS, vascular risk score; MMSE, mini-mental state examination.

a Adjusted for age, sex, education, APOE4, and VRS.

b Adjusted for age, sex, education, APOE4, VRS, BMI, annual income, physical activity, alcohol intake, smoking, albumin, fasting glucose, and HDL-/LDL-cholesterol.

### Investigation of the association between helplessness, subjective memory problem, and cognitive decline

The multiple linear regression analyses revealed that helplessness was associated with lower TS, with this association remaining significant even after adjusting for potential confounders (B = −5.300, SE = 1.899, *β* = −0.162, *p* = 0.006) (Table 7). Subjective memory problem was also associated with lower TS (B = −3.557, SE = 1.680, *β* = −0.109, *p* = 0.036) (Table 7, Model 1). However, this association ceased to be significant after adjusting for potential confounders (Table 7, Model 2).

**Table 7:** Results of multiple linear regression analyses of the associations between helplessness, subjective memory problems, and cognitive decline.

	Total score of CERAD			
	B	SE	*β*	*p*
*GDS item #10 (helplessness)*				
Model 1^a^				
Helplessness	−5.724	1.887	−0.175	0.003
Non-helplessness		Reference		
Model 2^b^				
Helplessness	−5.300	1.899	−0.162	0.006
Non-helplessness		Reference		
*GDS item #14 (subjective memory problems)*				
Model 1^a^				
Subjective memory problems	−3.557	1.680	−0.109	0.036
Non-subjective memory problems		Reference		
Model 2^b^				
Subjective memory problems	−3.177	1.667	−0.097	0.058
Non-subjective memory problems		Reference		

CERAD, Consortium to Establish a Registry for Alzheimer’s Disease; APOE4, apolipoprotein ε4; VRS, vascular risk score; BMI, body mass index.

a Adjusted for age, sex, education, APOE4, and VRS.

b Adjusted for age, sex, education, APOE4, VRS, BMI, annual income, physical activity, alcohol intake, smoking, albumin, fasting glucose, and HDL-/LDL-cholesterol.

### Influence of physical activity on the association between helplessness and cognitive decline

The analysis revealed a significant interaction between helplessness and physical activity, indicating that physical activity influences the relationship between hKelplessness and TS (B = 0.090, SE = 0.042, *β* = 0.225, *p* = 0.034). On the other hand, interactions between helplessness and other factors such as age, sex, APOE4, VRS, and BMI was not significant (Table 8). Further subgroup analysis indicated that helpless was significantly linked to TS only among participants engaging in lower level of physical activity (B = −7.291, SE = 2.862, *β* = −0.216, *p* = 0.013). However, this relationship was not observed in participants with higher level of physical activity (Table 9).

**Table 8:** Results of multiple linear regression analyses including helplessness × one covariate interaction term, predicting cognitive decline.

	Total score of CERAD			
	B	SE	*β*	*p*
Helplessness	−44.930	22.641	−1.375	0.049
Age	−0.637	0.205	−0.243	0.002
Helplessness × Age	0.546	0.311	1.224	0.081
Helplessness	−6.679	2.253	−0.204	0.003
Sex	−2.349	3.275	−0.069	0.474
Helplessness × Sex	4.748	4.183	0.094	0.258
Helplessness	−5.180	2.098	−0.158	0.015
APOE4	−5.938	2.691	−0.149	0.029
Helplessness × APOE4	−0.686	5.066	−0.010	0.892
Helplessness	13.848	13.284	0.424	0.299
BMI	0.531	0.338	0.115	0.118
Helplessness × BMI	−0.774	0.531	−0.592	0.147
Helplessness	−5.889	2.562	−0.180	0.023
VRS	0.077	0.074	0.080	0.299
Helplessness × VRS	0.039	0.115	0.032	0.732
Helplessness	−11.200	3.252	−0.343	<0.001
Physical activity	0.005	0.024	0.015	0.828
Helplessness × Physical activity	0.090	0.042	0.225	0.034

CERAD, Consortium to Establish a Registry for Alzheimer’s Disease; APOE4, apolipoprotein ε4; BMI, body mass index; VRS, vascular risk score.

**Table 9:** Results of multiple linear regression analyses of the associations between helplessness and cognitive decline according to physical activity status.

	Total score of CERAD			*p*
	B	SE	*β*	
*Low physical activity (n = 98)*				
Model 1^a^				
Helplessness	−7.627	2.802	−0.226	0.008
Non-helplessness	Reference			
Model 2^b^				
Helplessness	−7.291	2.862	−0.216	0.013
Non-helplessness	Reference			
*High physical activity (n = 98)*				
Model 1^a^				
Helplessness	−3.543	2.652	−0.114	0.185
Non-helplessness	Reference			
Model 2^b^				
Helplessness	−3.207	2.800	−0.103	0.255
Non-helplessness	Reference			

CERAD, Consortium to Establish a Registry for Alzheimer’s Disease; APOE4, apolipoprotein ε4; VRS, vascular risk score; BMI, body mass index.

a Adjusted for age, sex, education, APOE4, and VRS.

b Adjusted for age, sex, education, APOE4, VRS, BMI, annual income, alcohol intake, smoking, albumin, fasting glucose, and HDL-/LDL-cholesterol.

### Investigation of the association between overall GDS and MCI diagnosis

Multiple logistic regression analyses revealed that overall GDS contributes to the diagnostic accuracy for MCI (odds ratio = 1.071, 95% CI = 1.024 to 1.121, *p* = 0.003) (Supplementary Table S1).

### Impact of overall GDS alone and in combination with MMSE on MCI diagnosis accuracy

The Model G (incorporating overall GDS alone) exhibited high diagnostic accuracy for MCI (Supplementary Table S2). Furthermore, the MCI diagnostic accuracy of Model GM (combining overall GDS and MMSE) was significantly superior to that of Model M (Supplementary Table S2).

### Impact of helplessness alone and in combination with TS on MCI diagnosis accuracy

The Model H and Model T (incorporating TS alone) exhibited high diagnostic accuracy for MCI (Supplementary Table S2). Furthermore, the MCI diagnostic accuracy of Model HT (combining helplessness and TS) was significantly superior to that of Model T (Supplementary Table S3).

## Discussion

This study in nondemented adults was conducted to assess the relationship between individual components of depression within GDS items for diagnosing MCI and overall cognitive decline. It also sought to compare the diagnostic accuracy of a combined depression components and MMSE against the use of MMSE alone. Furthermore, the study explored the link between individual depression components and overall cognitive function, as indicated by the TS.

Our findings from logistic regression analyses of one-candidate models revealed that both ‘subjective cognitive problem item’ (GDS item #14) and MMSE exhibited diagnostic accuracy for MCI. Furthermore, analyses of two-candidate models demonstrated that the combination of ‘subjective cognitive problem item’ and MMSE displayed superior diagnostic accuracy for MCI compared to MMSE alone. These findings align with the results of prior studies (Tombaugh and McIntyre, 1992; Mackin et al., 2012; Choe et al., 2018; Arevalo-Rodriguez et al., 2021). A study demonstrated that the presence of subjective memory problems was equivalent to the MMSE in terms of detecting MCI or early AD (Choe et al., 2018). A longitudinal study with a 3-year follow-up period revealed that the experience of heightened subjective memory problems had a more significant influence than depressive symptoms in the progression to dementia (Mackin et al., 2012). In addition, the MMSE has reported limitations in clinical and research settings despite its extensive use in cognitive impairment screening (Tombaugh and McIntyre, 1992). Consequently, efforts have been made to enhance diagnostic accuracy by incorporating alternative scales (Breton et al., 2019) and additional tests (Xu et al., 2002). A review study has affirmed that relying solely on the MMSE for early detection of AD is not sufficient (Arevalo-Rodriguez et al., 2021). This has prompted research into more comprehensive approaches for improved cognitive assessments.

Interestingly, the present study showed that ‘helpless item’ (GDS item #10) alone and the combination of ‘helpless item’ and MMSE had the excellent accuracy for diagnosing MCI. The findings of the present study are indeed intriguing, revealing that ‘helpless item’ alone and the combination of ‘helpless item’ with MMSE displayed excellent accuracy in diagnosing MCI. This study introduces novel insights for addressing the limitations often associated with the use of MMSE in diagnosing MCI. The present study also demonstrated that helpless was significantly associated with overall cognition, with this association being moderated by physical activity. Notably, the cognitive decline related to helplessness was more evident in cases of lower physical activity, but could be alleviated through higher physical activity, bearing important clinical consequences. Collectively, these findings underline the importance of emotional factors, especially helplessness, in the context of AD and related cognitive decline. Such insights are crucial in refining cognitive assessments and could enhance strategies for early identification and prevention of conditions like MCI within the AD spectrum as well as in combating cognitive decline.

Feelings of helplessness is an emotional state where one perceives a lack of control over current life events and involve a lack of efforts to escape from uncontrolled stress (Grant, 1978; Ejdemyr et al., 2021; Song and Vilares, 2021). This differs from hopelessness, which is marked by a negative outlook on future outcomes (Ejdemyr et al., 2021). Feelings of helplessness are intimately linked to the psychological model of ‘learned helplessness’ as a framework for understanding depression, and exhibit high face, construct, and predictive validity in animal models of depression (Vollmayr and Gass, 2013). In animal experiments, loss of control over stress is frequently employed as depression models (Yao et al., 2019). It is established that more stress is experienced in situations where control is absent later on, compared to situations where there is no control from the beginning (Hanson et al., 1976). Concerning the mechanism underlying the relationship between helplessness and MCI or AD-related cognitive decline, feelings of helplessness arising from uncontrollable chronic stress are believed to contribute to the cumulative disturbance of homeostatic mechanisms. These mechanisms include the hypothalamic–pituitary–adrenal (HPA) axis, glucocorticoid receptor, and amyloid-beta or tau proteins. Consequently, uncontrollable chronic stress might be associated with an elevated risk of developing both depression and AD (Leonard, 2010; Kurakin and Bredesen, 2020; Saeedi and Rashidy-Pour, 2021; Sharma et al., 2021; Lyons et al., 2022). More specifically, alterations in the HPA axis, a significant contributor to depressive disorders, are linked to feelings of helplessness (Holsboer, 2000). Conversely, findings from an experimental study in mice propose that a reduction in glucocorticoid receptor expression is associated with sensitivity to stress and the development of learned helplessness (Ridder et al., 2005). Early damage to the HPA axis is recognized in AD, and abnormalities in glucocorticoid receptors have been associated with amyloid-beta pathology in AD animal models (Canet et al., 2020). There is a suggestion of a glucocorticoid response element in the promoter region of the gene encoding amyloid precursor protein, affecting the amyloid-beta pathway in response to stress (Lahiri, 2004). Additionally, excess glucocorticoids are known to impact tau phosphorylation (Yan et al., 2010). Collectively, feelings of helplessness arising from uncontrolled stressors may exert a shared influence on depressive disorders and AD. Their shared homeostatic dysfunctions suggest a potential link between helplessness and the pathogenesis of AD, possibly playing a pivotal role in the mechanisms of depression and AD.

## Strengths and limitations

This study is the first to demonstrate that the emotional component of depression, specifically helplessness, exhibits excellent diagnostic accuracy for MCI, either alone or in combination with MMSE. The findings remained robust even after adjusting for potential confounders such as biological, environmental, and blood nutritional biomarkers. The findings were also confirmed validated through sensitivity analysis, which involved excluding participants without moderate to extremely severe depressive symptoms. Additionally, the study revealed that helplessness was associated with lower overall cognition. This association was pronounced in condition of lower physical activity, but not in that of higher physical activity. From a clinical standpoint, the findings suggest potential utility in enhancing the diagnostic accuracy of MCI and AD-related cognitive decline compared to existing methods. Moreover, the cognitive decline associated with helplessness is more pronounced in instances of lower physical activity and can be offset by higher physical activity, which has significant clinical implications.

Nevertheless, our study had some limitations. The study’s cross-sectional nature prevented the confirmation of a causal relationship. To address potential reverse causality, we excluded individuals with severe medical conditions or moderate to extremely severe depression that could impact cognition. Since all participants had no severe medical conditions and were less severely depressed, reverse causality could not account for our observation of the relationship between helplessness and cognitive decline. Nevertheless, additional long-term follow-up studies are necessary to elucidate the causal relationships. While the study did not observe a significant link between other components of depression and the diagnosis of MCI or cognitive decline, this absence of correlation might be attributed to insufficient statistical power due to the size of the sample.

In conclusion, our research with older adults reveals that helplessness among individual depression components is highly effective in diagnosing MCI and is associated with reduced cognitive function. It is recommended that clinicians consider emotional factors like helplessness, in addition to MMSE and subjective memory problems, in assessing MCI and cognitive decline associated with AD.

## Data availability statement

The raw data supporting the conclusions of this article will be made available by the authors, without undue reservation.

## Ethic statement

The studies involving humans were approved by Institutional Review Board of Hallym University Dongtan Sacred Heart Hospital. The studies were conducted in accordance with the local legislation and institutional requirements. The participants provided their written informed consent to participate in this study.

## Author contributions

BL: Formal analysis, Methodology, Writing – original draft, Writing – review & editing. YC: Formal analysis, Methodology, Writing – original draft, Writing – review & editing. G-HS: Formal analysis, Methodology, Writing – original draft, Writing – review & editing. MK: Formal analysis, Methodology, Writing – original draft, Writing – review & editing. SK: Formal analysis, Methodology, Writing – original draft, Writing – review & editing. HK: Formal analysis, Methodology, Writing – original draft, Writing – review & editing. JH: Formal analysis, Methodology, Writing – original draft, Writing – review & editing. DY: Formal analysis, Methodology, Writing – original draft, Writing – review & editing. JK: Conceptualization, Data curation, Formal analysis, Funding acquisition, Investigation, Methodology, Supervision, Writing – original draft, Writing – review & editing.

## Footnotes

^1^http://www.law.go.kr
