# Supplementary material for: Implications of helplessness in depression: diagnosing mild cognitive impairment and analyzing its effects on cognitive decline in older adults
Source: Front Aging Neurosci. 2024 May 27;16:1378676. doi: 10.3389/fnagi.2024.1378676 (PMC11163037; doi:10.3389/fnagi.2024.1378676)
Supplement: Supplementary file 1 [file Table_1.pdf]

SUPPLEMENTARY MATERIALS

Table S1 Multiple logistic regression analyses of overall GDS score for MCI diagnosis.

Table S2 Logistic regression analyses to select appropriate models for MCI diagnosis

Table S3 Logistic regression analyses to select appropriate models for MCI diagnosis

This supplementary material has been provided by the authors to give readers additional information about their work.

Table S1. Multiple logistic regression analyses of overall GDS score for MCI diagnosis.

	OR	95% CI	*P*	R^2
Overall GDS score				
Model 1 [a]	1.066	1.020 to 1.113	0.004	0.154
Model 2 [b]	1.071	1.024 to 1.121	0.003	0.215

GDS geriatric depression scale, MCI mild cognitive impairment, APOE4 apolipoprotein E ε4 allele, VRS vascular risk score, BMI body mass index.

[a] Adjusted for age, sex, education, APOE4, and VRS.

[b] Adjusted for age, sex, education, APOE4, VRS, BMI, annual income, physical activity, alcohol intake, smoking, albumin, fasting glucose, and HDL-/LDL-cholesterol.

Table S2. Logistic regression analyses[a] to select appropriate models for MCI diagnosis

	Classification accuracy (%)	-2LL	χ^2	*P*	Δ *df*	Significance test for -2LL difference
One candidate model						
Model G						
Overall GDS	70.1	230.389	33.895	0.003	1	
Model M						
MMSE	71.1	213.314	50.970	<0.001	1	
Two candidate model						
Model GM						
Overall GDS + MMSE	72.7	204.706	59.578	<0.001	1	*Model GM vs. M:*<br>*P* = 0.003<br>△ accuracy (%) = 1.6

MCI mild cognitive impairment, GDS geriatric depression scale, MMSE mini-mental state examination.
-2LL indicates -2 log likelihood.
[a] Adjusted for age, sex, education, APOE4, VRS, BMI, annual income, physical activity, alcohol intake, smoking, albumin, fasting glucose, and HDL-/LDL-cholesterol.

Table S3. Logistic regression analyses [a] to select appropriate models for MCI diagnosis

	Classification accuracy (%)	-2LL	χ^2	*P*	Δ *df*	Significance test for -2LL difference
One candidate model						
Model H						
GDS item #10	70.1	220.967	43.316	<0.001	1	
Model T						
TS	82.5	149.706	114.578	<0.001		
Two candidate model						
Model HT						
GDS item #10 + TS	86.6	141.641	122.643	<0.001	1	*Model HT vs. T:* *P* = 0.005 △ accuracy (%) = 4.1

MCI mild cognitive impairment, GDS geriatric depression scale, MMSE mini-mental state examination.
-2LL indicates -2 log likelihood.
[a] Adjusted for age, sex, education, APOE4, VRS, BMI, annual income, physical activity, alcohol intake, smoking, albumin, fasting glucose, and HDL-/LDL-cholesterol.
